# Integrative metabolome and transcriptome analyses provide insights into PHGDH in colon cancer organoids

**DOI:** 10.1042/BSR20240842

**Published:** 2025-01-28

**Authors:** Lin Chen, Zhihui Dai, Yanfei Zhang, Huichao Sheng, Bin Hu, Jinlin Du, Jie Chang, Wenxia Xu, Yuqing Hu

**Affiliations:** 1 Central Laboratory, Precision Medicine Center, Affiliated Jinhua Hospital, Zhejiang University School of Medicine, Jinhua, Zhejiang Province, 321000, China; 2 Jinhua Key Laboratory of Cancer Nutrition and Metabolism Research, Affiliated Jinhua Hospital, Zhejiang University School of Medicine, Jinhua, Zhejiang Province, 321000, China; 3 Department of Colorectal Surgery, Affiliated Jinhua Hospital, Zhejiang University School of Medicine, Jinhua, Zhejiang Province, 321000, China; 4 Department of General Surgery, The Second Affiliated Hospital of Soochow University, Suzhou, Jiangsu, 215006, China; 5 Department of Pathology, Affiliated Jinhua Hospital, Zhejiang University School of Medicine, Jinhua, Zhejiang Province, 321000, China

**Keywords:** colon cancer organoids, metabolome, PHGDH, transcriptome

## Abstract

As a rate-limiting enzyme in the endogenous serine *de novo* synthesis pathway, 3-Phosphoglycerate dehydrogenase (PHGDH) has been widely concerned about its role in a variety of tumors including colon cancer and the development of inhibitors. In our previous study, we studied PHGDH in colon cancer cell lines. However, with the development of personalized therapy, we realized that in scientific research, two-dimensional cell lines lost a lot of original characteristic information during long-term culture, and the results obtained may not be enough to support the conclusion. Patient-derived tumor organoids maintain genomic stability and make up for information missing from cell lines due to monoclonal growth. Therefore, in our study, a colon cancer organoid with high PHGDH expression was selected and analyzed for transcriptomic and metabolomic changes through targeted inhibition of PHGDH. The results showed that inhibition of PHGDH significantly inhibited the proliferation of colon cancer organoids. The transcriptome, metabolome, and combined omics analysis showed that the changes in colon cancer organoids after inhibition of PHGDH were mainly involved in PRSS1 and PRSS56, steroid hormone biosynthesis, phenylalanine metabolism, ascorbate and aldarate metabolism, and tyrosine metabolism. In our study, the role of PHGDH in serine metabolism in colon cancer organoids was clarified by multi-omics analysis to provide new knowledge for an in-depth understanding of serine metabolism and PHGDH function in colon cancer.

## Introduction

Colon cancer is one of the most common malignant tumors of the digestive tract. Surgery, chemoradiotherapy, targeted therapy, immunotherapy, and other strategies can prolong the survival of patients with colon cancer. However, the mechanisms involved in the occurrence, drug resistance, and metastasis of colon cancer still need to be deeply explored to provide a scientific basis for further improving the therapeutic effect.

In recent years, more and more attention has been paid to the role of metabolic reprogramming in tumorigenesis and development. The requirements of carbohydrates, amino acids, lipids, vitamins, and trace elements in tumor cells were significantly different from those in normal cells [1,2]. The rapid proliferation of tumor cells requires the synthesis of many biological macromolecules, such as proteins and nucleic acids, whose carbon source is derived from one-carbon units. Serine is the main source of one-carbon units, and tumor cells ensure the supply of serine through various ways, such as the intake of exogenous serine through high expression of amino acid transporters [3]. When exogenous serine is insufficient, tumor cells initiate *de novo* synthesis pathways that convert glucose into serine, thereby increasing endogenous serine supply. 3-Phosphoglycerate dehydrogenase (PHGDH) is the key rate-limiting enzyme in this process [4–8]. In gliomas [9] and cervical [10], pancreatic [11], and colorectal cancer [12], the overexpression of PHGDH is associated with advanced TNM stage, large tumor, higher tumor grade, and shorter overall survival time, respectively. High levels of PHGDH display rapid proliferation and migration [13]. Serine metabolic reprogramming is closely related to the progression of colon cancer. However, due to the lack of research models that accurately simulate tumors *in vivo*, the strategy of interfering with serine metabolizing enzymes such as PHGDH is still insufficient for anti-tumor therapy.

Organoid is a three-dimensional culture model that has been developed in recent years. The tissue-derived tumor organoids not only have a highly similar structure to the source tissues but also have a high degree of consistency in their response to drugs. Organoids have broad application prospects in the fields of drug sensitivity evaluation, mechanism study, biomarker screening and so on. Colon cancer is one of the earliest cancers to carry out organoid culture, and the success rate of organoid culture of colon cancer is relatively high. Several research groups have established biobanks of colon cancer organoids for drug screening, evaluation, occurrence and development research of colon cancer [14–17]. However, studies on serine metabolic reprogramming and PHGDH function based on the organoid technology platform of colon cancer have not yet been carried out.

Previously, we used multi-omics techniques to investigate the role of PHGDH in the serine metabolism of two-dimensional cultured colon cancer cells [18]. We also conducted the culture of colon cancer organoids and carried out the metabolomics analysis technique based on the organoid platform [19]. In the present study, we analyzed the transcriptomic and metabolomic changes in colon cancer organoids by targeting inhibition of PHGDH. The results showed that inhibition of PHGDH significantly inhibited the proliferation of colon cancer organoids. Transcriptome and metabolome data analysis showed that the organoid changes after inhibition of PHGDH involved the imbalance and compensation of amino acid metabolism, lipid metabolism, glucose metabolism, and chromatin modification. Finally, based on a combined analysis of transcriptome and metabolome, we focused on the PRSS1 and PRSS56, steroid hormone biosynthesis, phenylalanine metabolism, ascorbate and aldarate metabolism, and tyrosine metabolism. In conclusion, this study provides a new way to further understand the mechanism of serine metabolic reprogramming and precise intervention strategies for colon cancer.

## Materials and methods

### Patient-derived organoid culture

Colon cancer patient-derived organoids (PDOs) were established in previous studies and evaluated for morphological and genomic characteristics [19]. Methods of organoid culture for colon cancer have been described in previous studies [19]. In simple terms, organoids were resuspended in growth factor reduced (GFR) Matrigel: DMEM/F12K (1:1, v/v) mixture, and seeded in a well of a 24-well flat bottom cell culture plate (Corning) preheated at 37°C. The Matrigel was then solidified by a 20-minute incubation in a 37 ℃ and 5% CO_2_ cell culture incubator and overlaid with 500 µl of complete human colon cancer organoid medium (Shanghai Woomiao Biotechnology Co., LTD, WMH-03). PDO medium was subsequently refreshed every 3 days.

All experimental protocols were approved by the Medical Ethics Committee of Jinhua Hospital of Zhejiang University.

### PDO drug assays

PDOs were harvested and dissociated into single cells following the passaging procedure described above. Cell pellets were resuspended in 500 µl of PDO medium WMH-03. Cells were counted with the Countess Automated Cell Counter (Thermo Fisher Scientific), 10 µl of GFR Matrigel: PDO medium (1:1, v/v) mixture containing 3,000 cells were seeded in standard 96-well cell culture plates (Corning). The Matrigel was solidified by a 20-minute incubation in a 37 ℃ and 5% CO_2_ cell culture incubator, then 100 μl of PDO medium was added to each well to continue the culture. The medium was removed after 3 days of seeding plates and replaced with 100 µl of PHGDH inhibitor (NCT-503)-containing PDO medium and cultured for 7 days at 37℃ in the 5% CO_2_ atmosphere. The PDO medium was subsequently refreshed every 3 days. The organoids under the same field of view were captured under the same magnification microscope (10× eyepiece, 10× objective lens) in three fields on days 0, 4, and 7, respectively. The captured organoids were measured using the OPLENIC_Pro software to measure the organoid mass diameter (μm). Organoid mass with a diameter of 20 ± 5 μm on day 0 was selected, and the mass diameter of the same mass was tracked and measured on day 4 and 7. The measurements are mapped statistically.

After 7 days of NCT-503 intervention, 10 μL CCK-8 reagent (Beyotime, Shanghai, China) was added to each well and cultured in 5% CO_2_ for 1 h continuously. The optical density (OD) value was measured by a microplate reader (Synergy HT ZX-22; Bio-Tek Instruments, USA) at a wavelength of 450 nm.

### Metabolomic analyses

#### Sample preparation of cell lysate for metabolomic analyses

The PDOs were treated with IC50 concentrations of NCT-503 (57.61 μM) for 7 days. The medium was gently aspirated and cells on the surface of the well plates were gently washed with ice-cold PBS (phosphate buffer saline) three times. The GFR Matrigel and PDO mixtures were dispersed with 1 ml of ice-cold PBS and gently blown evenly. The suspension was transferred to a centrifuge tube at 300 *g*/min and centrifuged at 4℃ for 5 min. The supernatant was discarded and the previous step was repeated three times to wash away the remaining GFR Matrigel. The centrifugal tubes containing the PDO precipitate were contacted with liquid nitrogen to quench the cells. QC samples are made by mixing the samples to be measured in equal quantities and distributed in the front, middle, and end of the detection queue. Metabolites in samples were detected based on HPLC-Orbitrap MS/MS. Electrospray ionization (ESI) in the mass spectrometer was conducted in positive and negative modes in full scan.

#### Data preprocessing and metabolite identification

The metabolites were identified by matching with information such as retention time, molecular mass and secondary spectra of metabolites in local databases. To better conduct subsequent data analysis, the raw data are further cleaned. A single feature was filtered to retain peak area data with no more than 50% missing values in the experimental sample. Individual features were filtered to remove background noise. Peak area data with relative standard deviation (RSD) greater than 30% in QC samples were removed. The missing value recoding in the data was simulated. The method was 10% of the minimum value. The obtained high-quality features were annotated according to the HMDB database (http://www.hmdb.ca). Due to limitations, we did not use standards for verification. Pathway analysis was performed using MetaboAnalyst 5.0 (http://www.MetaboAnalyst.ca/).

### RNA-sequencing analysis

TRIzol reagent (Carlsbad, CA, USA) was used to extract total RNA from PDOs treated with NCT-503 (57.61 μM) for 7 days. Three independent replicates were analyzed. RNA-sequencing (RNA-seq) was performed by KAITAI-BIO (Hangzhou, China). RNA-seq libraries were constructed using the Illumina TruSeq RNA sample preparation kit (RS-122–2001) and sequenced using an Illumina high-seq 2000 with a read length of 50 bp with pair[20] . Only those reads mapped to unique genomic locations and with <5% mismatches were analyzed further. We used FPKM [21] to measure gene transcript expression and DEGSeq [22] to identify differentially expressed genes. The differentially expressed genes were counted and annotated using the NCBI, UniProt, KEGG, and GSEA databases to obtain detailed descriptive information.

### Immunohistochemistry (IHC) staining

Tumor biopsies were fixed with 4% paraformaldehyde (PFA, Sigma Aldrich) at 4°C overnight, dehydrated and embedded in paraffin, and then sectioned onto microscope slides. Organoids with Matrigel were generally removed from the culture plate and fixed in 4% PFA at room temperature overnight followed by polymerizing into agarose gel. These deparaffinized CRC tissues and CRC organoid sections were subsequently subjected to IHC staining. To analyze CRC-PDO phenotypes, the following antibodies were used: (1) anti-PHGDH antibody (1:1000, catalog number: 14719–1-AP, Proteintech, USA), (2) anti-Ki67 antibody (1:1000, catalog number: MX006, Maixin, China). Calculated the number of cells with positive expression of Ki67 in 200 cells in a random field of view. Differences were determined by the parametric unpaired Sstudent’s *t*-test between two groups via GraphPad Prism software (San Diego, California, USA), and *P*<0.05 was considered statistically significant.

### Combined analysis of transcriptome and metabolome

To further understand the mechanism of PHGDH in colon cancer organoids, the transcriptome and metabolome were comprehensively analyzed by expression correlation analysis, KEGG co-enrichment analysis. Expression correlation analysis and KEGG enrichment analysis were performed using Metware Cloud, a free online platform for data analysis (https://cloud.metware.cn).

## Results

### The expression level of PHGDH in colon cancer PDOs was consistent with the parental tumor

**Figure 1:**
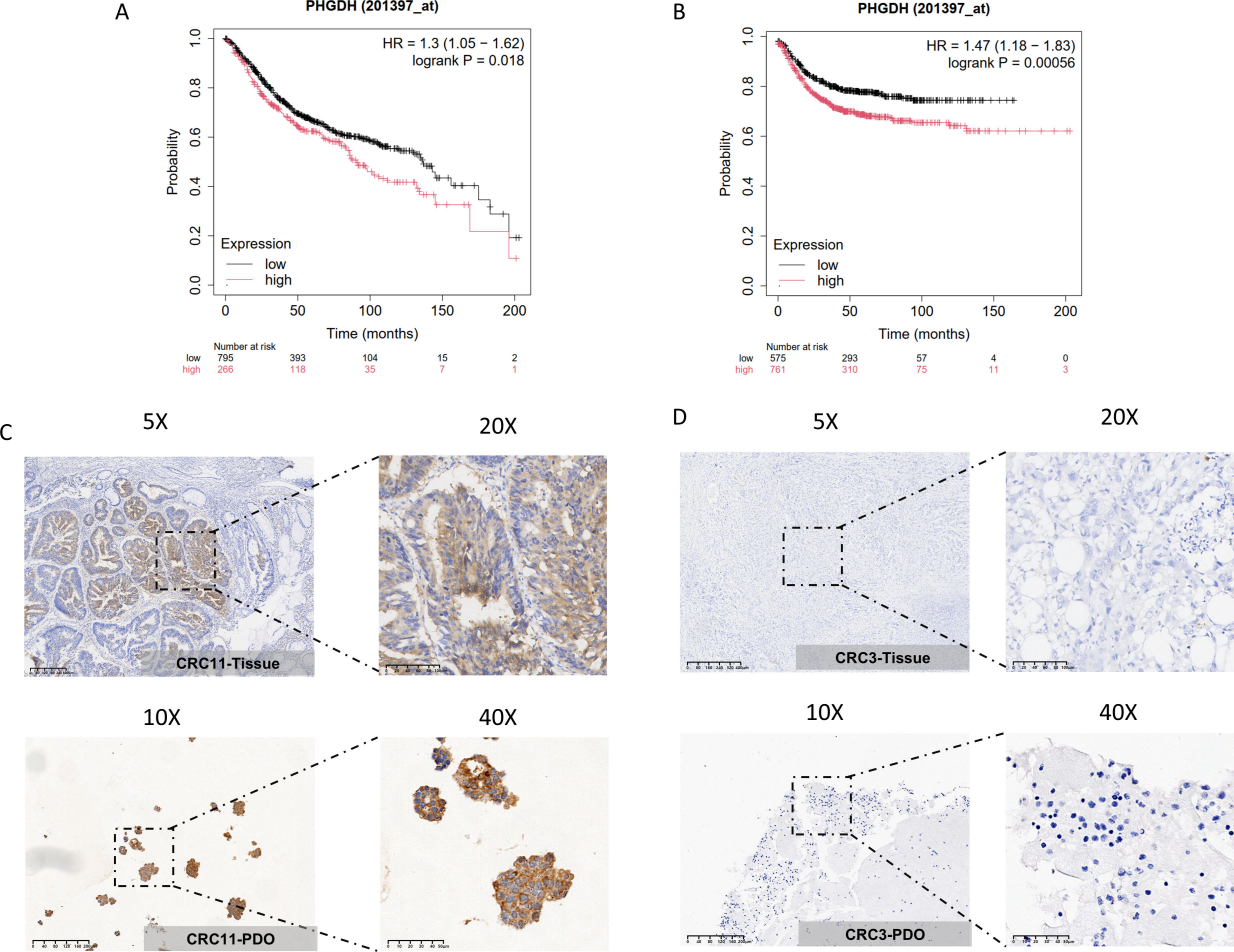
The expression level of PHGDH in colon cancer patients and PDOs. (**A**) Relationship between PHGDH expression and overall survival of colon cancer patients, which was measured by Kaplan –Meier plotter (*P*<0.05). (**B**) Relationship between PHGDH expression and recurrence -free survival of colon cancer patients, which was measured by Kaplan –Meier plotter (*P*<0.05). IHC staining of CRC tissues and their corresponding organoids. Pathological marker for IHC staining was PHGDH. (**C**) PHGDH expression in CRC11-PDO and parental tissue. (**D**) PHGDH expression in CRC3-PDO and parental tissue. Scale bar of 5×, 400 μm; scale bar of 10×, 100 μm; scale bar of 20×, 200 μm; scale bar of 40×, 50 μm.

The structure and morphology of organoids are highly consistent with the drug reactivity of tumors *in vivo*. Therefore, it is more realistic to study the function of PHGDH based on colon cancer organoids. In previous study, we constructed colon cancer organoids and confirmed the high consistency between these colon cancer organoids and the parental tissues through morphological analysis and gene mutation spectrum detection [19]. To investigate the prognostic value of PHGDH in colon cancer, survival analysis was measured in colon cancer patients by Kaplan–Meier plotter. Colon cancer patients with higher PHGDH expression were associated with shorter overall survival (Figure 1A) and recurrence-free survival (Figure 1B) (*P*<0.05). Therefore, it is more appropriate to select colon cancer organoids with high PHGDH expression as research objects. In this study, we first tested the expression of PHGDH in six parental tissues (Supplementary Figure S1). Immunohistochemical tests showed that the expression of PHGDH in colon cancer organoids numbered CRC11 and its parental tissues was strongly positive (Figure 1C), while the expression of PHGDH in colon cancer organoids numbered CRC3 and its tissue of origin was negative (Figure 1D). These results indicated the consistency of PHGDH expression in colon cancer organoids and their parental tissues.

### The PHGDH inhibitor NCT-503 inhibits the proliferation of colon cancer PDOs

**Figure 2:**
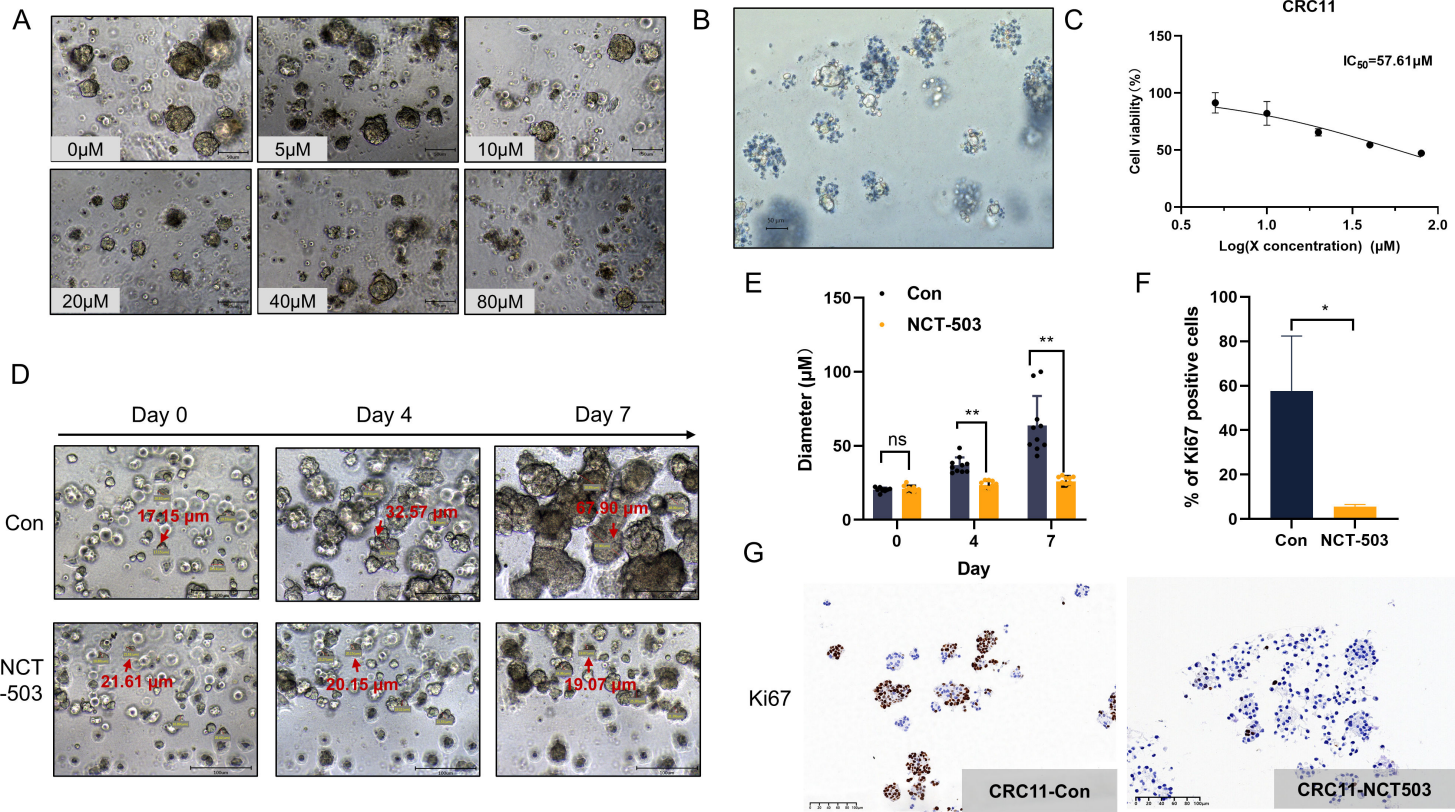
Drug response of CRC PDOs to NCT-503. After 0 μM, 5 μM, 10 μM, 20 μM, 40 μM, and 80 μM NCT-503 were administered in different CRC PDOs for 7 days, the morphology of PDO recorded under the microscope (**A**), and the comparison of cell activity detected by CCK8-kit (**C**). Scale bar, 50 μm. (**B**) Morphology of organoids after administration with 0.4% Trypan blue staining. Scale bar, 50 μm. (**D**) Morphology of CRC11 on days 0, 4, and 7 under the administration of 57.61 μM NCT-503. (**E**) Diameter statistics of CRC11 on days 0, 4, and 7 under the administration of 57.61 μM NCT-503. *N* = 10. (**F-G**) IHC staining and statistical analysis of CRC11. Ki67 expression in CRC11-Con and CRC-NCT503. Scale bar, 100 μm.

The organoid CRC11 with positive PHGDH expression was selected as the study object. NCT-503 is a selective inhibitor of PHGDH that selectively blocks serine synthesis from glucose sources [23]. We applied NCT-503 to CRC11 to observe the effect of inhibiting PHGDH in colon cancer. The untreated organoids had dense structure and smooth edges. With the increase in the concentration of NCT-503, the edges of organoid gradually loosened (Figure 2A, Supplementary Figure S2A). By staining with 0.4% trypan blue, the loose edges of the clusters were blue, and the central masses were uncolored (Figure 2B). These results suggested that the stereo structural changes caused by inhibition of PHDGH are a heterogeneous death. According to the CCK-8 test, the median inhibitory concentration (IC_50_) of NCT-503 in CRC11 was 57.61 μM (Figure 2C). The CellTiter-Blue® Cell Viability Assay was used to verify again, and similar results were obtained (Supplementary Figure S2B).

To quantify the inhibitory effect of NCT-503 on CRC11, organoids with a diameter of 20 ± 5 μm were selected and their diameter was measured at days 0, 4, and 7 of NCT-503 (IC_50_). The diameters of the control group continued to increase, and the edges were tight with budding. On the contrary, the mass diameters of the NCT-503 treatment group showed a decreasing trend (Figure 2D and E). Furthermore, the area (integration) of organoids treated with NCT503 (IC_50_) was detected by an integrated fluorescence microscopy imaging system (KEYENCE, BZ-X800), and the results showed that the overall growth of the NCT503 treatment group slowed down (Supplementary Figure S2C). This conclusion was re-verified by recording the overall growth status of organoids after treatment with different concentrations of NCT503 on days 0, 4, and 7 (Supplementary Figure S2D). The method of diameter and area measurements directly reflected the inhibitory effect of targeting PHGDH on colon cancer organoids. In order to further verify the inhibitory effect of NCT-503 on the proliferation of colon cancer organoids, CRC11 was prepared into paraffin sections after 7 days of NCT-503 treatment. The expression of Ki67 was detected by immunohistochemistry. As shown in Figure 2F and G, Ki67 was significantly expressed in the CRC11-Con group, with an expression rate as high as 60%, indicating that tumor cells had a strong proliferation capacity. On the contrary, the expression of Ki67 was significantly decreased after treatment with NCT-503. This suggested that NCT-503 can indeed inhibit the proliferation of PHGDH-dependent colon cancer organoids.

### Metabolomics analysis of PHGDH inhibition in colon cancer PDOs

**Figure 3:**
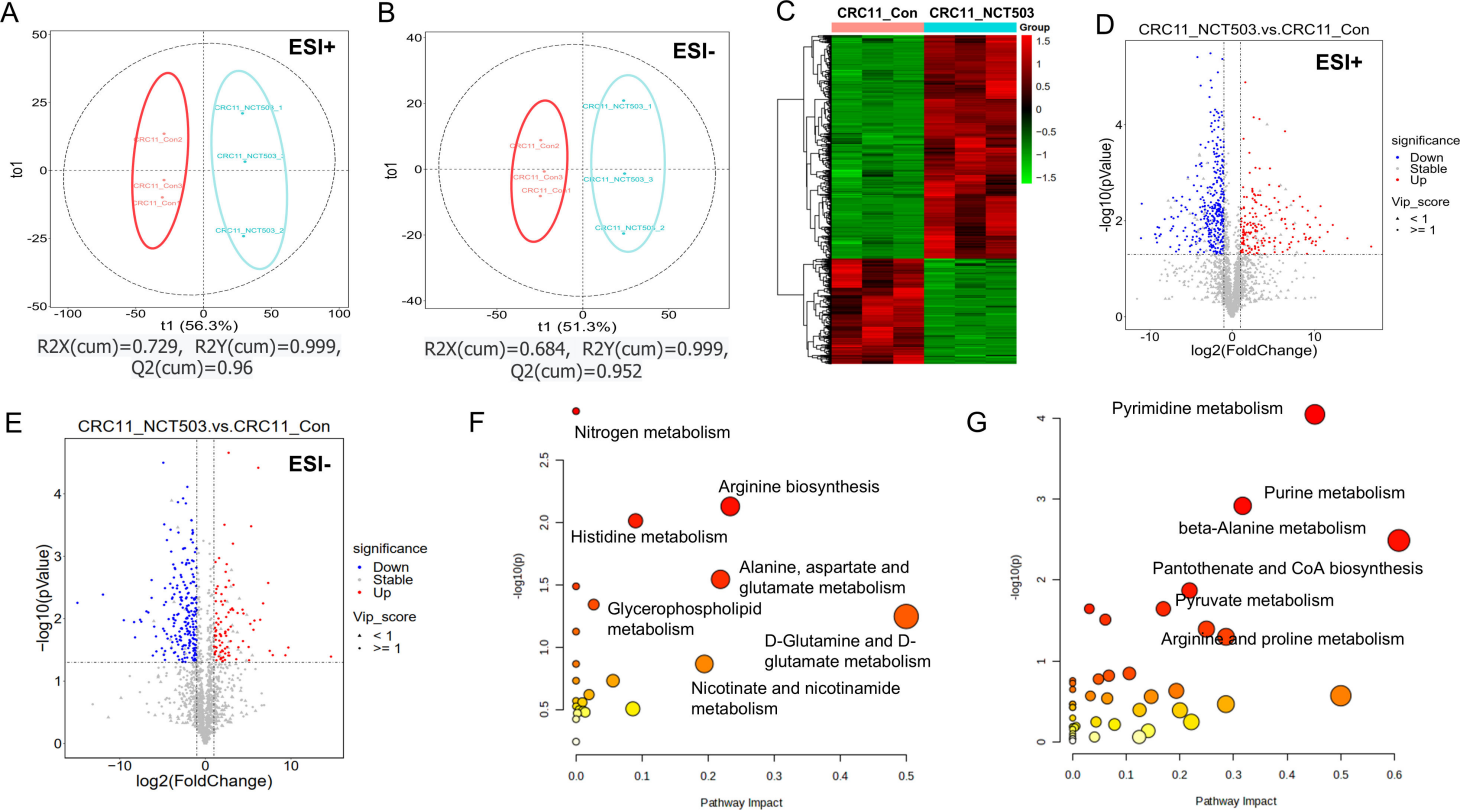
HPLC- MS/MS metabolic profiles of CRC11. (**A**) OPLS-DA score plot of positive ion mode (ESI+) in CRC11-Control (CRC11-Con), CRC11-NCT-503 (CRC11-NCT503) groups. R2X(cum) = 0.729, R2Y(cum) = 0.999, Q2(cum) = 0.96. (**B**) OPLS-DA score plot of negative ion mode (ESI-) in CRC11-Con and CRC11-NCT503 groups. R2X(cum) = 0.684, R2Y(cum) = 0.999, Q2(cum) = 0.952. The Rred circle corresponds to CRC11-Con group, blue pentagon corresponds to CRC11-NCT503 group. (**C**) Heat map showed 518 metabolites that were significantly different between CRC11-Con and CRC11-NCT503 groups in positive and negative mode. (**D**) Volcano plot of positive ion mode (ESI+) and negative ion mode (ESI-) (**E**) in CRC11-Con and CRC11-NCT503 groups. (**F**) Metabolic pathways whichthat were enriched by up-regulated metabolites in CRC11-NCT503 compared towith CRC11-Con. (**G**) Metabolic pathways which that were enriched by down-regulated metabolites in CRC11-NCT503 compared to with CRC11-Con. In the bubble plot, Y-axis represents enriched metabolic pathways, X-axis means pathway impacts. The size of each bubble represents the number of metabolites enriched and the color indicates the p*P* -value (take the negative natural logarithm). Small p *P*-value and big pathway impact factor revealed that the pathway is significantly affected.

Inhibition of PHGDH leads to obstruction of endogenous serine synthesis, leading to metabolic remodeling of cells. We previously performed metabolomics and transcriptomic analyses of two-dimensional cultured colon cancer cells after targeted inhibition of PHGDH. In order to better understand the response of colon cancer to PHGDH inhibition, we used colon cancer PDOs to analyze the metabolomics and transcriptomic changes after PHGDH inhibition. The metabolites of CRC11 after treatment with NCT-503 were detected by liquid chromatography-tandem mass spectrometry (LC-MS). Partial least squares model (OPLS-DA) analysis showed that treatment with NCT-503 resulted in significant changes in the metabolic profile of PDOs in both positive and negative ion modes (Figure 3A and B). Heat maps (Figure 3C) and volcanic maps (Figure 3D and E) showed that the number of metabolites changed after treatment with NCT-503. A total of 518 metabolites had their contents changed, of which 150 were up-regulated and 368 were down-regulated (Supplementary Table S1). The top ten metabolites that were most up-regulated were deoxycytidine, 2-methoxyestrone 3-glucuronide, 5-megastigmen-7-yne-3,9-diol 9-glucoside, 1,2,4-nonadecanetriol, LysoPC(22:1/0:0), (13Z)-docos-13-enoylcarnitine, taurochenodeoxycholate-3-sulfate, pipercitine, persin, and LysoPC(24:1/0:0). The top ten metabolites that were most significantly down-regulated were oleoylcarnitine, L-2-aminoadipate adenylate, tetradecanoylcarnitine, tetradecanal, N-formyl-L-methionine, pentadecanal, N1-acetylspermine, 2-[(methylthio)methyl]-2-butenal, bethanechol, and pyroglutamic acid. KEGG enrichment analysis revealed that the metabolic pathways composed of up-regulated metabolites were mainly nitrogen metabolism, arginine biosynthesis, histidine metabolism, alanine, aspartate and glutamate metabolism, glycerophospholipid metabolism, D-glutamine and D-glutamate metabolism, nicotinate, and nicotinamide metabolism (Figure 3F). The main metabolic pathways composed of down-regulated metabolites were pyrimidine metabolism, purine metabolism, beta-alanine metabolism, pantothenate and CoA biosynthesis, pyruvate metabolism, arginine, and proline metabolism (Figure 3G). We further focused on levels of some of the metabolites involved in these pathways. After intervention with NCT-503, most metabolites in the glycerophospholipid pathway were significantly up-regulated, especially lysophospholipid (Supplementary Figure S3A,B). Alanine, uracil, and histidine levels in beta-alanine metabolism were significantly down-regulated (Supplementary Figure S3C). At the same time, argininosuccinic acid and L-glutamic acid were significantly up-regulated in the arginine-related pathway, and ornithin, N2-acetylornithine, phosphocreatine, and 4-guanidinobutanoic acid were significantly down-regulated (Supplementary Figure S3D).

### Transcriptomic analysis of PHGDH inhibition in colon cancer PDOs

**Figure 4:**
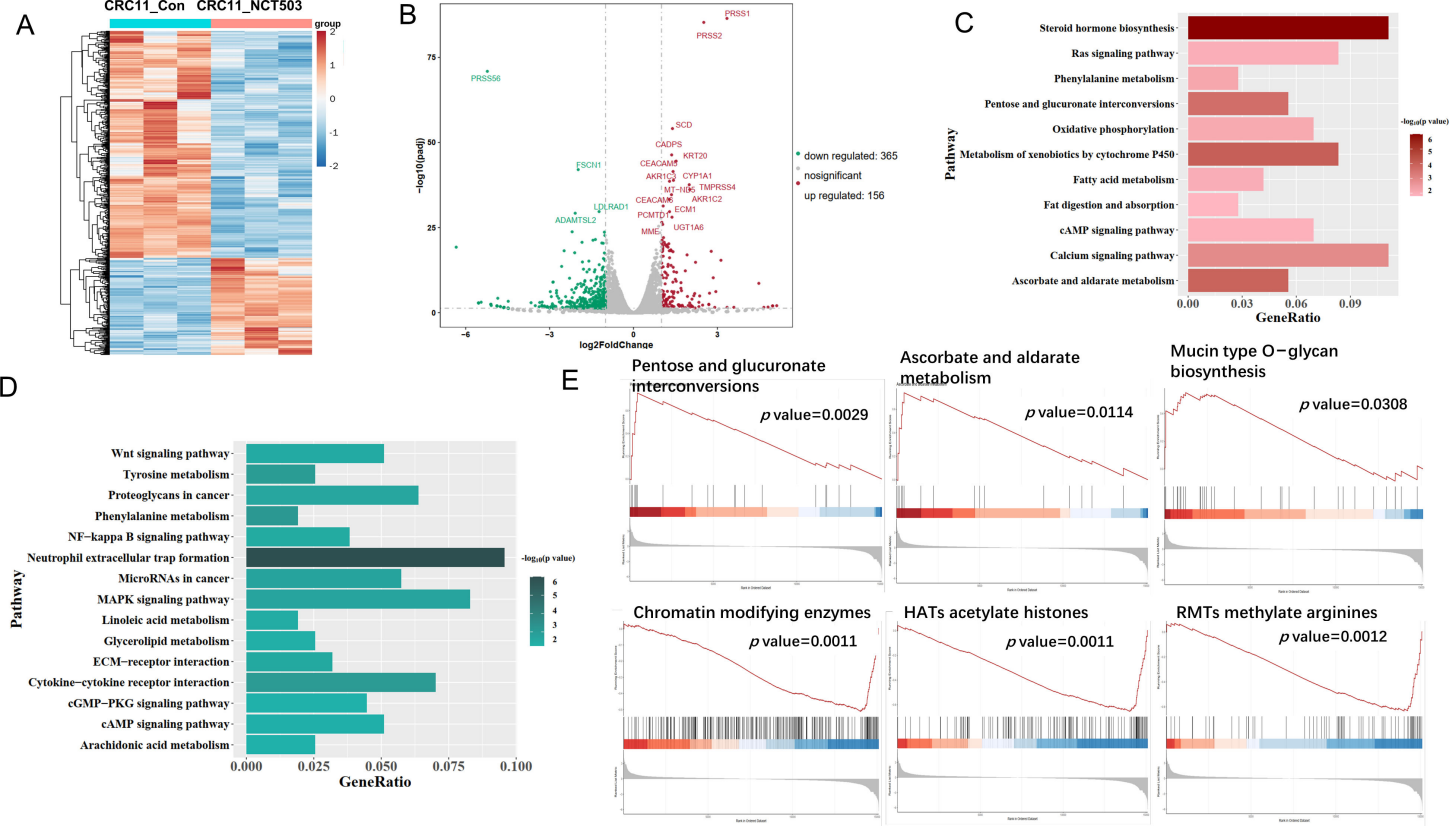
Transcriptomic analysis of PHGDH inhibition in CRC11. (**A**) Heat map showed 521 genes that were most significantly different between CRC11-Con and CRC11-NCT503 groups. (**B**) Volcano plot illustrating the differently expressed genes in CRC11-Con and CRC11-NCT503 groups. (**C**) KEGG annotations and enrichment of up-regulated genes in CRC11-NCT503 compared to with CRC11-Con.(**D**) KEGG annotations and enrichment of down-regulated genes in CRC11-NCT503 compared to with CRC11-Con. (**E**) Gene set enrichment analysis (GSEA) of RNA-seq data in CRC11-Con and CRC11-NCT503 groups.

To clarify the effect of targeted inhibition of PHGDH on the expression of colon cancer organoids, we performed a transcriptomic analysis of NCT-503 treated CRC11. The results showed that the expression of 521 genes was changed after treatment with NCT-503 (Figure 4A and B), among which 156 up-regulated genes and 365 down-regulated genes were expressed (Supplementary Table S2). The top ten genes that were most up-regulated were NLRP6, CC2D2B, UGT1A3, LIN7A, IQGAP2, TENM4, FAM50B, LMOD1, PRSS1, and MSMB. The top ten most significantly down-regulated genes were EPHA8, TNFSF14, CCL2, GHRL, ZSCAN4, PRSS56, ADAMTS2, EVC, ROBO4, and CHRND. KEGG enrichment analysis showed that the signaling pathways composed of up-regulated genes were mainly steroid hormone biosynthesis, ascorbate and aldarate metabolism, pentose and glucuronate interconversions (Figure 4C). The signaling pathways of down-regulated metabolites are mainly neutrophil extracellular trap formation, phenylalanine metabolism, and tyrosine metabolism (Figure 4D). Gene set enrichment analysis (GSEA) further found that pentose and glucuronate interconversions, ascorbate and aldarate metabolism, and mucin type O−glycan biosynthesis signaling pathway are significantly up-regulated, while chromatin modifying enzymes, HATs acetylate histones, and RMTs methylate arginines (arginine methyltransferases) were significantly down-regulated (Figure 4E). These results suggested that the transcriptomic changes in colon cancer PDOs after inhibition of PHGDH were involved in oxidative stress, glucose metabolism, and amino acid metabolism.

### Combined analysis of transcriptome and metabolome

**Figure 5:**
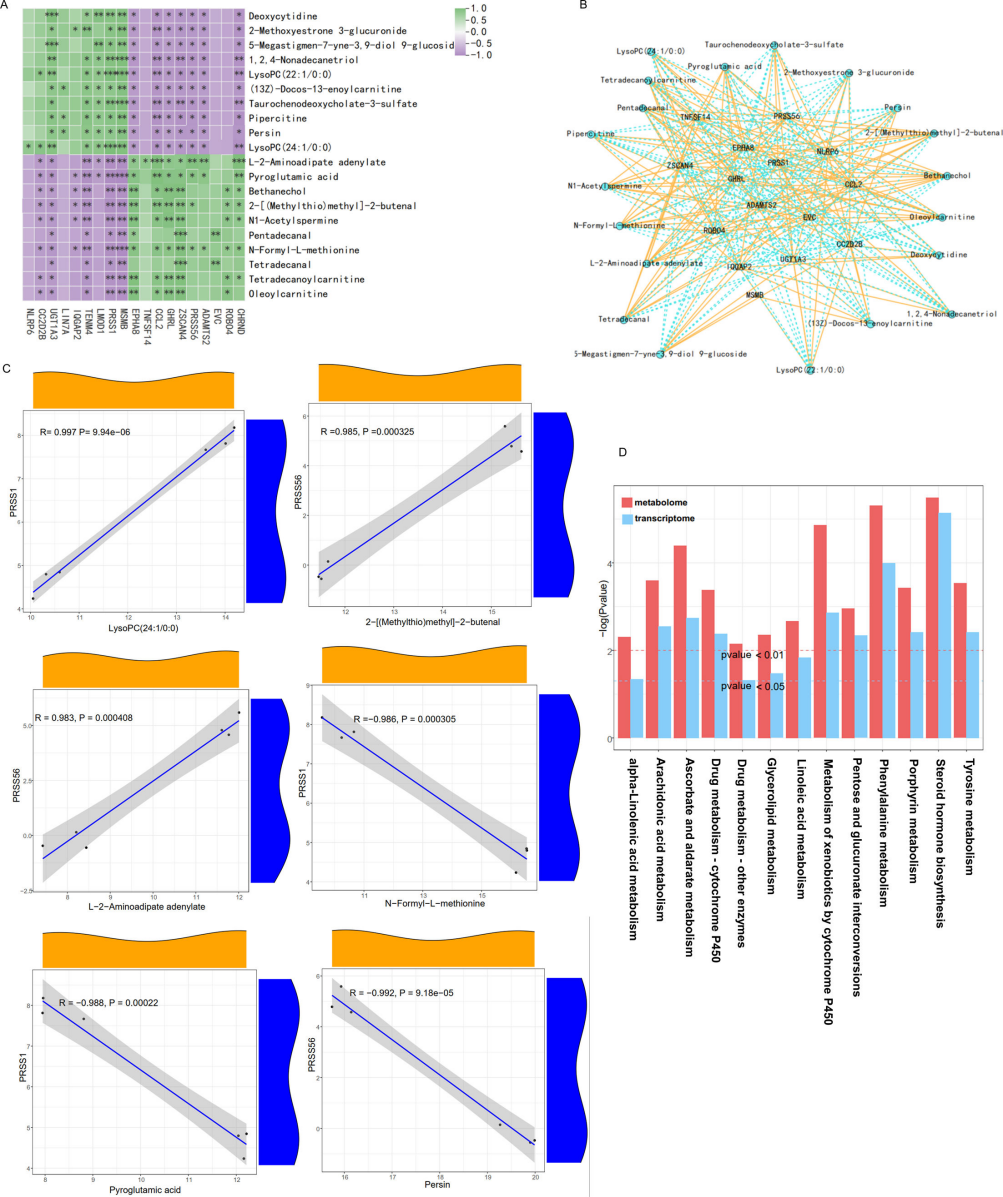
Joint transcriptomic and metabolomic correlation analysis. (**A**) Correlation heat map of the top 20 differentially expressed genes and metabolites (10ten up-regulated and 10 ten down-regulated) between CRC11-Con and CRC11-NCT503 groups. (**B**) Relevance network diagram of the top 20 differentially expressed genes and metabolites. (**C**)Smooth correlation scatter plots of the first six pairs of metabolites and genes. (**D**) Joint KEGG pathway co-enrichment of transcriptome and metabolome.

The combined analysis of transcriptome and metabolome is based on the standard analysis results of the two omics, and the correlation analysis of the annotation results of differential genes and differential metabolites in metabolic pathways can better explain the transcriptional regulatory mechanisms in metabolic pathways. To further understand the mechanism of PHGDH on colon cancer organoids, the transcriptome and metabolome were comprehensively analyzed by expression correlation analysis, KEGG co-enrichment analysis in this study. Correlation analysis of the top 20 differentially expressed genes and metabolites (ten up-regulated and ten down-regulated) between the two groups showed that the correlation between differentially expressed genes and metabolites was high (Figure 5A). Differential gene and differential metabolite association network maps were plotted according to Pearson correlation coefficient > 0.9 and *P*-value < 0.05 (Figure 5B). It indicated that the changes in differentially metabolites regulating were significantly related to the changes in differentially expressed genes. The first six pairs of metabolites and genes were mapped with smooth correlation scatter plots. LysoPC (24:1/0:0) is positively correlated with PRSS1, 2-[(Methylthio)methyl]-2-butenal, and L-2-aminoadipate adenylate is positively correlated with PRSS56. Pyroglutamic acid, N-formyl-L-methionine were negatively correlated with PRSS1, and persin was negatively correlated with PRSS56 (Figure 5C). Joint KEGG pathway co-enrichment was performed, resulting in 13 co-enriched pathways, among which steroid hormone biosynthesis was the most significantly regulated pathway, followed by phenylalanine metabolism, ascorbate and aldarate metabolism, and tyrosine metabolism (Figure 5D).

## Discussion

Amino acid metabolic reprogramming plays an important role in the occurrence and development of various tumors. Therefore, targeted amino acid metabolism strategies have become one of the hot spots in anti-tumor research [24]. The expression levels of many amino acid metabolic enzymes were significantly increased in tumors, such as LAT1, ASS1, PHGDH, PSAT1, etc. [25–29]. Studies on the expression and function of amino acid metabolizing enzymes will help improve anti-tumor strategies of amino acid metabolism restriction.

As a rate-limiting enzyme in the endogenous serine *de novo* synthesis pathway, PHGDH has been widely concerned about its role in a variety of tumors and the development of inhibitors [30–34]. Studies have reported that PHGDH protein is highly expressed in colon cancer [35] and has a significant negative correlation with the prognosis of colon cancer [12,36].

In our previous study, we analyzed the gene expression and metabolic remodeling process of colon cancer cells after targeted inhibition of PHGDH by gene transcriptomics and metabolomics and found that amino acid transporters, amino acid metabolism, lipid synthesis-related pathways compensation, and other processes are involved in the response process after PHGDH inhibition [18]. However, with the development of personalized therapy, we realized that in scientific research, two-dimensional cell lines lost a lot of original characteristic information during long-term culture, and the results obtained may not be enough to support the conclusion. Therefore, in this study, patient-derived colon cancer organoids were treated with PHGDH-specific inhibitors to further understand the function of PHGDH. Our study confirms for the first time that the selective PHGDH inhibitor NCT-503 has a significant proliferative inhibitory effect on colon cancer organoids.

Compared with previous findings in colorectal cancer cells [18], lipid metabolism, amino acid metabolism, and purine metabolism were also enriched in colon cancer organoids with high PHGDH expression. The difference was that genes and pathways related to arginine metabolism are highly enriched in intestinal cancer organoids. Through joint omics analysis, we noticed PRSS1 and PRSS56, which were members of the trypsin family of serine proteases. PRSS1 is active on peptide linkages involving the carboxyl group of lysine or arginine [37]. Li et al. found that PRSS56 is reactivated in cancers by promoter DNA hypomethylation and PRSS56 functions oncogenic roles in colon and gastric cancer by activating PI3K/AKT axis [38]. In this study, after NCT-503 intervention in CRC11, PRSS56 was down-regulated and the proliferation of CRC11 was inhibited. Results of KEGG pathway co-enrichment suggested that steroid hormone biosynthesis, phenylalanine metabolism, ascorbate and aldarate metabolism, and tyrosine metabolism changed significantly. PHGDH inhibition could reduce the incorporation into nucleotides of one-carbon units from glucose-derived and exogenous serine [23]. We think that the intervention of NCT-503 leads to metabolic reprogramming of intestinal cancer organoids.

It has been reported that inhibiting serine synthesis can inhibit tumor growth [29,35,39]. Tong et al. found that a serine/glycine-free diet inhibited colon cancer growth and enhanced anti-tumor immunity, and clinical trials proved that a serine/glycine-free diet was feasible and safe [40]. Gong et al. found that the anticancer properties of the selective PHGDH inhibitor CBR-5884 in epithelial ovarian cancer cells exhibiting high PHGDH expression, manifesting through the suppression of proliferation, migration, and invasion, while enhancing chemotherapy sensitivity [41]. Therefore, targeted inhibition of PHGDH to inhibit serine metabolism is expected to become a new strategy for the treatment of colon cancer. NCT-503 has good absorption, distribution, metabolism, and excretion (ADME) characteristics. It was reported that NCT-503 attenuated the growth of PHGDH-dependent cell lines both in culture and in orthotopic xenograft tumors [23]. Dong et al. used NCT-503 to overcome erlotinib resistance in EGFR mutation-positive lung adenocarcinomas [42]. Wang et al. used NCT-503 to induce ferroptosis and overcome enzalutamide resistance in castration-resistant prostate cancer cells [43]. These findings suggest that PHGDH-targeted inhibitors combined with chemotherapy may provide a new strategy for the treatment of colon cancer. In future studies, we can screen drug-resistant organoid strains in our laboratory’s colon cancer organoid bank, and try to combine NCT-503 or CBR-5884 with various chemotherapy regimens to explore new strategies of PHGDH inhibitors in the treatment of colon cancer.

All in all, in this study, we selected colon cancer organoids with high PHGDH expression, analyzed the transcriptomic and metabolomic changes through targeted inhibition of PHGDH, and found that inhibition of PHGDH significantly inhibited the proliferation of colon cancer organoids. The transcriptome, metabolome, and combined omics analysis showed that the changes in colon cancer organoids after inhibition of PHGDH were mainly involved in PRSS1 and PRSS56, steroid hormone biosynthesis, phenylalanine metabolism, ascorbate and aldarate metabolism, and tyrosine metabolism. In conclusion, this study provides a new way to further understand the mechanism of serine metabolic reprogramming and precise intervention strategies for colon cancer.

## Supplementary material

Online supplementary figure 1

Online supplementary figure 2

Online supplementary figure 3

Uncited online supplementary material 1

Online supplementary table 1

Online supplementary table 2

## Data Availability

All supporting data are included within the main article and its supplementary files. The metabolomic data are available and can be downloaded from Supplementary Material 1. The transcriptomic data are available and can be downloaded from Supplementary Material 2.

## Abbreviations

ADME: Absorption, distribution, metabolism, and excretion
ESI: electrospray ionization
GFR: growth factor reduced
GSEA: gene set enrichment analysis
IC50: median inhibitory concentration
IHC: immunohistochemistry
LC-MS: liquid chromatography-tandem mass spectrometry
OD: optical density
PBS: phosphate buffer saline
PDOs: patient-derived organoids
PFA: paraformaldehyde
PHGDH: 3-phosphoglycerate dehydrogenase
RSD: relative standard deviation
