# Supplementary figures and images for: Integrative metabolome and transcriptome analyses provide insights into PHGDH in colon cancer organoids
Source: Biosci Rep. 2025 Jan 28;45(1):BSR20240842. doi: 10.1042/BSR20240842 (PMC12096955; doi:10.1042/BSR20240842)

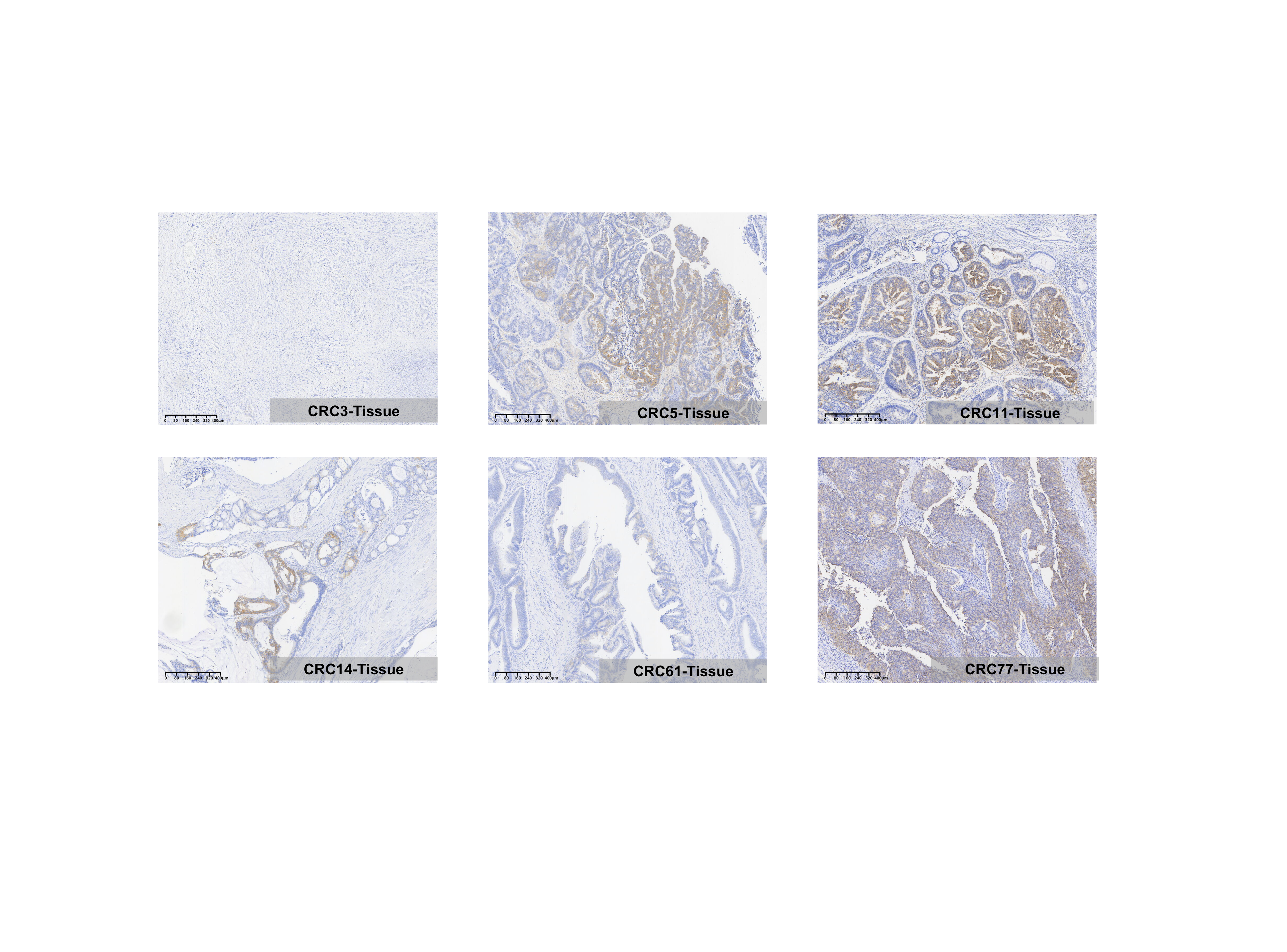

Supplement: Online supplementary figure 1 [file bsr-45-01-bsr-2024-0842-s001.tif]

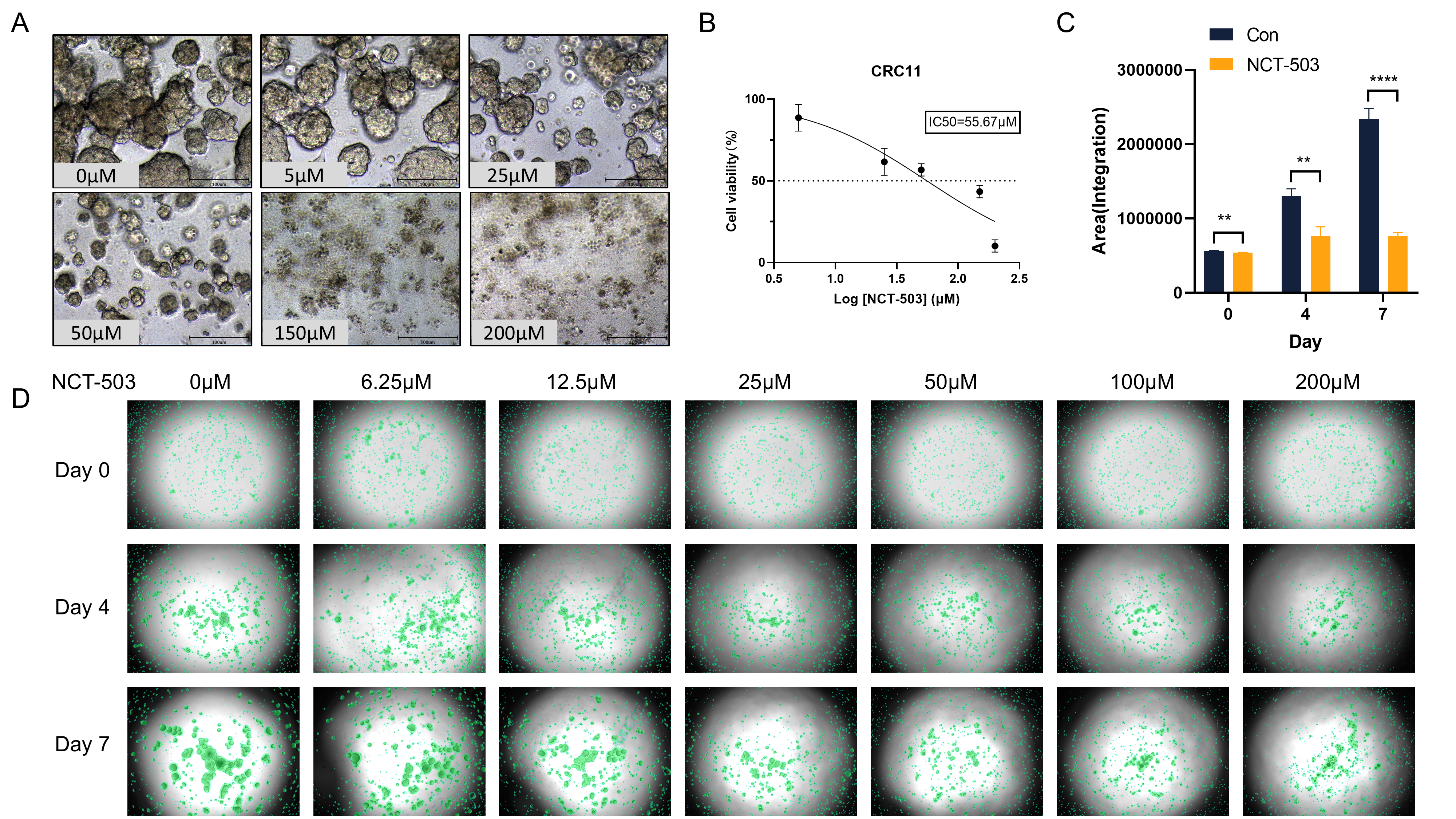

Supplement: Online supplementary figure 2 [file bsr-45-01-bsr-2024-0842-s002.tif]

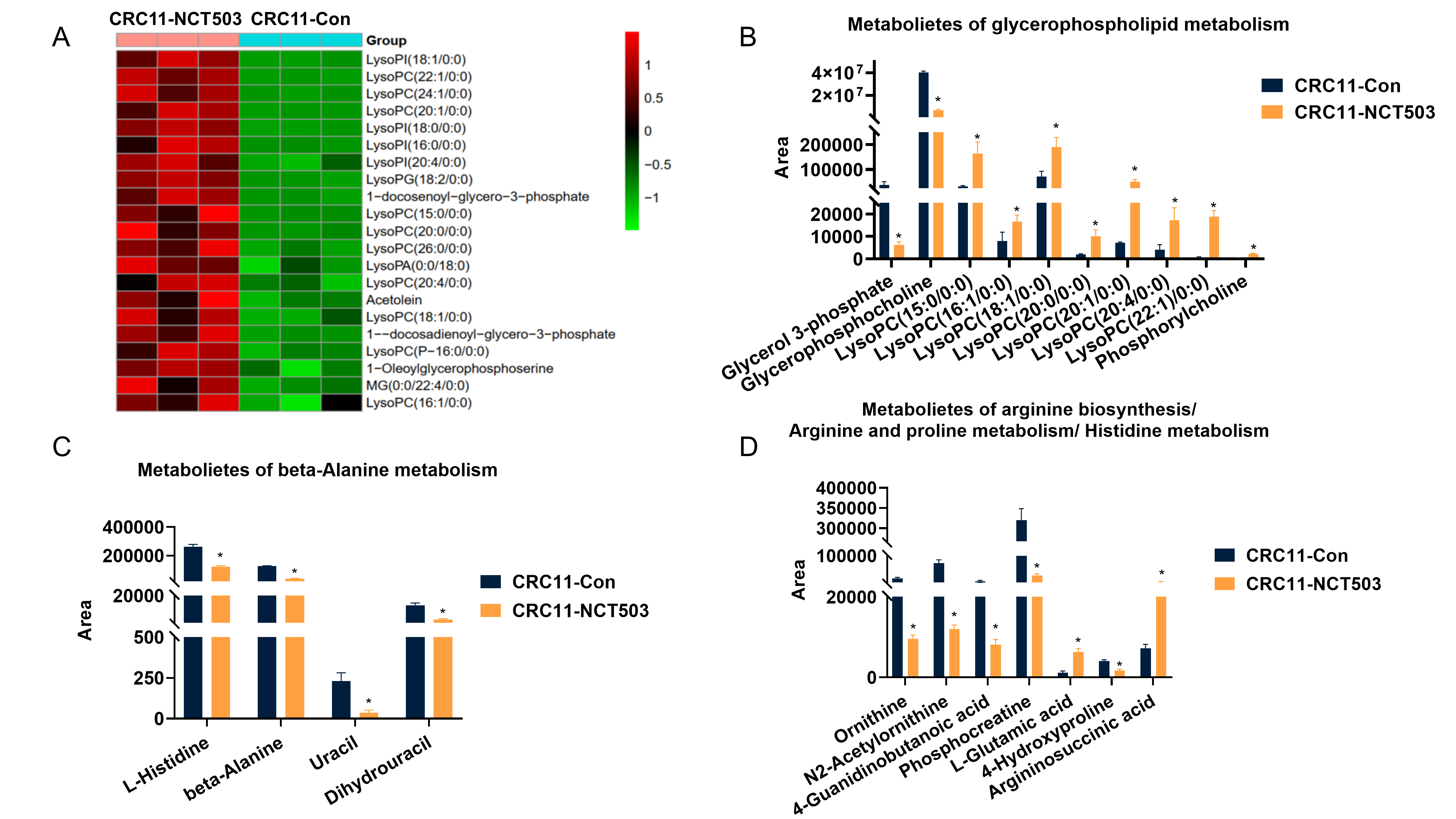

Supplement: Online supplementary figure 3 [file bsr-45-01-bsr-2024-0842-s003.tif]
