# Supplementary material for: Integrative metabolome and transcriptome analyses provide insights into PHGDH in colon cancer organoids
Source: Biosci Rep. 2025 Jan 28;45(1):BSR20240842. doi: 10.1042/BSR20240842 (PMC12096955; doi:10.1042/BSR20240842)

**Supplementary materials**

**Integrative Metabolome and Transcriptome Analyses Provide Insights into PHGDH in Colon Cancer Organoids**

Lin Chen^1，2#^, Zhihui Dai^3，4#^, Yanfei Zhang^5^, Huichao Sheng^5^, Bin Hu^5^ ,Jinlin Du^2^, Jie Chang^1，2^, Wenxia Xu^1，2*^, Yuqing Hu^1，2*^

1. Central Laboratory, Precision Medicine Center, Affiliated Jinhua Hospital, Zhejiang University School of Medicine, Jinhua, 321000, Zhejiang Province, China.
2. Jinhua Key Laboratory of Cancer Nutrition and Metabolism Research, Affiliated Jinhua Hospital, Zhejiang University School of Medicine, Jinhua, 321000, Zhejiang Province, China.
3. Department of Colorectal Surgery, Affiliated Jinhua Hospital, Zhejiang University School of Medicine, Jinhua 321000, Zhejiang Province, China.
4. Department of General Surgery, The Second Affiliated Hospital of Soochow University, Suzhou, China.
5. Department of Pathology, Affiliated Jinhua Hospital, Zhejiang University School of Medicine, Jinhua 321000, Zhejiang Province, China.

*^*^* Corresponding authors:[yqhu721@163.com](mailto:yqhu721@163.com), [xuwenxia@zju.edu.cn](mailto:xuwenxia@zju.edu.cn)

# These authors contributed equally to this work.

Cytotoxicity Assay Protocol

PDOs were harvested and dissociated into single cells following the passaging procedure described above. Cell pellets were resuspended in PDO medium WMH-03. Cells were counted with the countess automated cell counter (Thermo Fisher Scientific). 80 µl of cell suspension containing 3,000 cells were seeded in Ultra-Low Attachment Black 96-Well Plates with Clear Flat Bottom (BeyoGold™) and were cultured for 3 days at 37℃ in the 5% CO_2_ atmosphere. 20 µl of PHGDH inhibitor (NCT-503)-containing PDO medium were added after 3 days, and cultured for 7 days at 37℃ in the 5% CO_2_ atmosphere. After 7 days, removed assay plates from 37°C incubator and add 20µl/well of CellTiter-Blue® Reagent (Promega, G8080, USA). Shaked for 10 seconds and incubate using standard cell culture conditions for 4 hours. Then, shaked plate for 10 seconds and record fluorescence at 560/590nm.

Supplementary figure 1. PHGDH expression in six parent tissues of CRC PDOs. Scale bar of 5×, 400μm.

Supplementary figure 2. Drug response of CRC PDOs to NCT-503. (A-B) CRC PDO was treated with 0 μM, 5 μM, 25 μM, 50 μM, 150 μM and 200 μM NCT-503 for 7 days, and the morphology of PDO was recorded under the microscope (A), and the comparison of cell activity detected by CellTiter-Blue ® Cell Viability Assay (B). (C) Area (integration) statistics of CRC11 on day 0, 4 and 7 under the administration of 55.67μM NCT-503 . （D) After 0 μM, 6.25μM, 12.5 μM, 25μM, 50μM, 100μM and 200μM NCT-503 were administered in different CRC PDOs for 0, 4 and 7 days, the morphology of PDO recorded under the microscope .

Supplementary figure 3. (A) The expression levels of lipid metabolites in CRC11-Con and CRC11-NCT503 groups. (B) The expression levels of metabolites in glycerophospholipid metabolism pathway. N=3. (C) The expression levels of metabolites in beta-Alanine metabolism pathway. N=3. (D) The expression levels of metabolites in arginine biosynthesis, arginine and proline metabolism and histidine pathway. N=3. The ordinate of B, C, D represent the peak area of the metabolite in the mass spectrum.

Supplementary table 1 Identification of differential metabolites in CRC11-NCT503 group vs CRC11-Con group.

Supplementary table 2 Identification of differential genes in CRC11-NCT503 group vs CRC11-Con group.

Supplementary figure 1

**
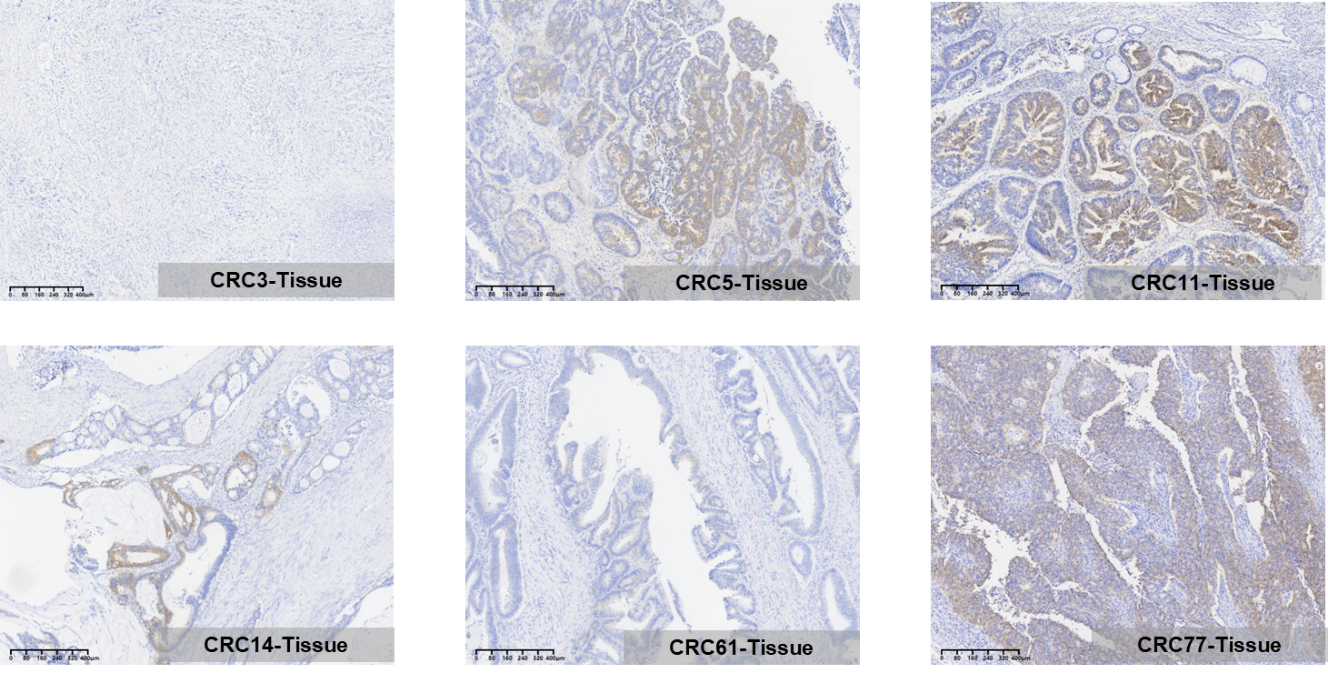
**

Supplementary figure 2


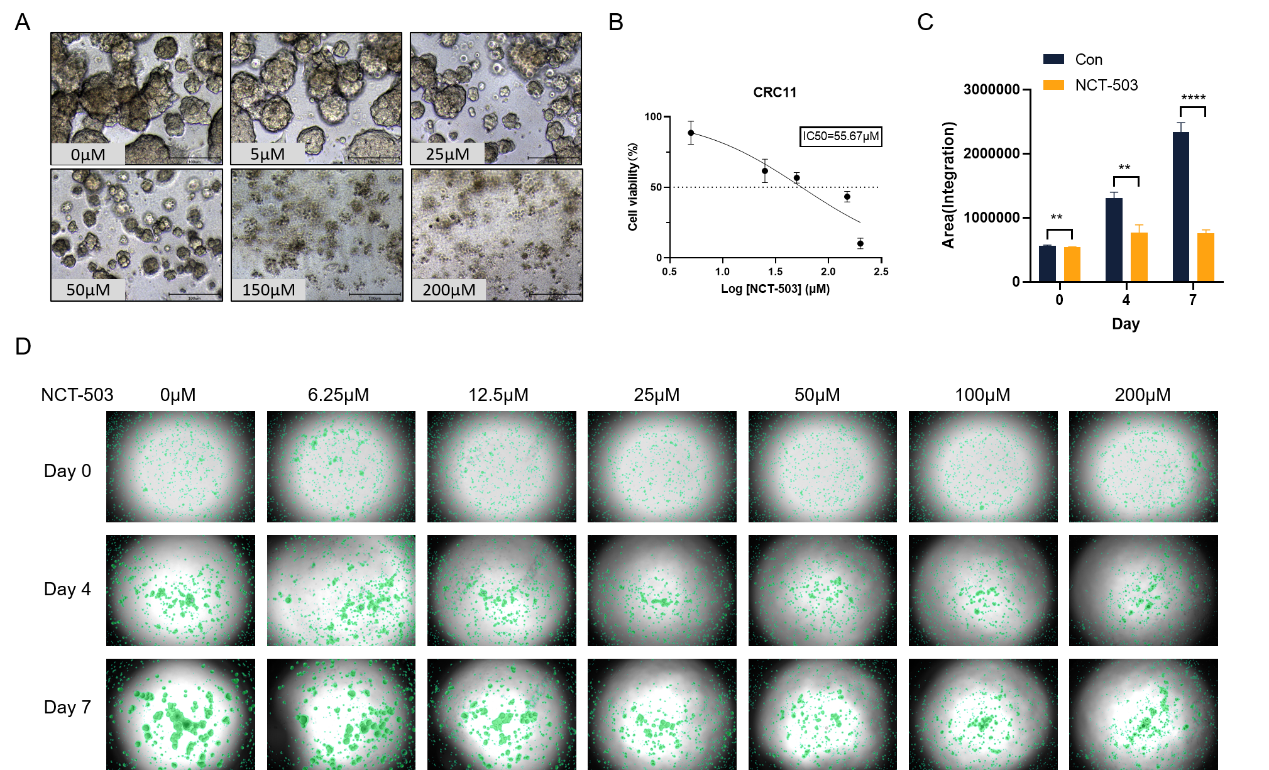


Supplementary figure 3


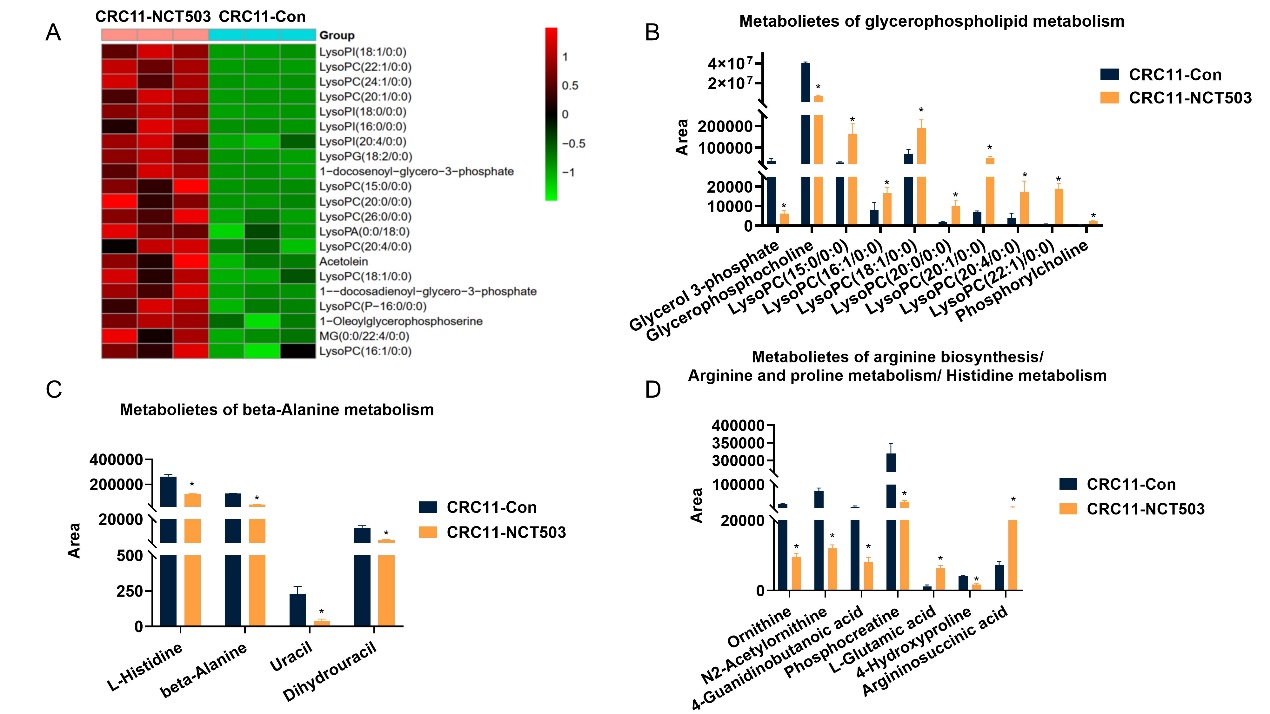

Supplement: Uncited online supplementary material 1 [file bsr-45-01-bsr-2024-0842-s004.docx]
